# Supplementary material for: Tongue strength and endurance among typically developing children and children with idiopathic speech sound disorders in the United Arab Emirates
Source: PLoS One. 2023 Jul 31;18(7):e0289400. doi: 10.1371/journal.pone.0289400 (PMC10389734; doi:10.1371/journal.pone.0289400)
Supplement: Attachment — Submitted filename: response_to_comments.docx [file pone.0289400.s001.docx]

Dear editor,

Thanks for considering our paper for publication. As you can see, the paper has been edited and we are confident that all grammatical errors have been corrected. The changes made have been highlighted in the revised manuscript. Below is our point-by-point response to the reviewers and editor’s comments.

| Comments | Response | Page number |
| --- | --- | --- |
| Editor |  |  |
| Based on the comments from Reviewer 1 and Reviewer 2, the decision as the academic editor would be to request a major revision of the manuscript. | Thanks once again for your feedback on our submission. |  |
| Reviewer 1 recommended accepting the manuscript. They found the research technically sound, supported by appropriate data and statistical analysis. They also highlighted the importance of the study in adding normative data to the database and encouraged further research in this area. No major concerns or competing interests were mentioned. | Thanks once again for your feedback on our submission |  |
| Reviewer 2, on the other hand, recommended a major revision. They acknowledged the novelty and relevance of the research but raised several issues. They pointed out that research question 2 was not fully answered in the results section, and there were inconsistencies in the writing quality, particularly in the discussion. Reviewer 2 also provided minor comments regarding clarity and phrasing in different sections of the manuscript. | Yes, we have paid attention to feedback from reviewer 2 and incorporate suggestions in our revised submission. |  |
| Considering the major comments and the need for clarification and improvement in certain areas of the manuscript, it would be appropriate to request a major revision from the authors. They should address the concerns raised by Reviewer 2, make necessary revisions to improve the writing quality and clarity, and ensure that research question 2 is appropriately addressed in the results section. Once the revisions are made, the manuscript can be re-evaluated for potential acceptance. | Yes, we have paid attention to feedback from reviewer 2 and incorporate suggestions in our revised submission. |  |
| Be sure to:  • Indicate which changes you require for acceptance versus which changes you recommend  • Address any conflicts between the reviews so that it's clear which advice the authors should follow  • Provide specific feedback from your evaluation of the manuscript | Thanks once again. We have indicated the changes made in our revised submission below. |  |
|  |  |  |
| Reviewer 1 |  |  |
| Reviewer #1: - The assessment of tongue strength and endurance and their effects on swallowing and speech forms an important component of the comprehensive evaluation of speech and language  - this study would be responsible for adding important normative data to the database of standardized measurements for maximal strength and endurance scores in the pediatric population of the United Arab Emirates. Future research is encouraged for the collection of additional data that can help healthcare professionals in objectively evaluating children with feeding, swallowing, and speech sound production difficulties. | Thanks once again for your feedback on our submission. |  |
| **Reviewer 2** |  |  |
| The present manuscript describes the first normative study on tongue strength and endurance in a paediatric population in the UAE. The novelty is well described in the introduction, and the relevance of this research to clinical practise is well explained in the introduction and the conclusion. | Thanks once again for your feedback on our submission. |  |
| This is a small study which could have benefited from a larger sample size, as disclosed by the authors who recommended that further larger studies confirm the results. In terms of writing quality, there is some inconsistency in the style and quality throughout the manuscript; while the introduction and methods section are relatively well written, the quality declines in the discussion. this would merit reviewing for English writing to improve some parts of the manuscript. i have mentioned a couple of sentences in the minor comments. | Yes, we can confirm that the discussion has been revised and edited thoroughly. | See pages 16-17 |
| At the end of the introduction, the authors clearly stated their research questions which makes it clear for the reader what the main outcomes of the study are. However, research question number 2 is not fully answered in the results section. it appears that what was measured was the association between sex, age, and type of child, but not the association with tongue strength and endurance. If this is correct, research question 2 should not include these variables. | Thanks for this. We have revised the research questions for clarity:  1. What are the tongue strength and endurance levels of TD children and children with ISSDs in the Arabian context?  2. Are there differences between participants across child type (TD vs. ISSDs), sex, and age on tongue strength and tongue endurance?  3. Are there associations between child type (TD vs. ISSDs), age, and sex? | See page 5 |
| - In the methods section, sub-section Participants, the description of the groups is a bit confusing. it is understood that 36 TD children were included in the study and all of them were diagnosed with ISSD. See below from page 12:  "Thirty-nine TD children were recruited to participate in this study; however, three of them did not cooperate during the data collection sessions and did not therefore complete the protocol. None of them had a history....... All of them were identified as having ISSDs by a blinded SLP and were enrolled in or had undergone speech therapy. " and then the authors go on to explain in the Procedures that the participants in this group (the TD group) were recruited from rehabilitation centers. No mention here of the 29 children in the ISSD group. | Thanks very much for this. We have detailed the participants who were recruited for this study. In page 6, we have indicated that 65 children were recruited, consisting of 36 typically developing and 29 children with ISSD. | See page 6 |
| - In the methods section, the fact that the instrumentation section is in between Participants description and recruitment affects the flow of the paper. It may be clearer to move the instrumentation section to after the procedure section or to move the recruitment information to the participants section. | Thanks for this. We have moved the procedure above. | See pages 7 |
| In the methods section, the authors should provide a rationale for their sample size calculation. Especially given the 2 groups and additional stratification of the data. Is a sample size of 65 children sufficient for normative test data? what about for group comparisons? | Yes, the sample size was sufficient. We have added the sample size calculation method used for this study. | See page 7 |
| - In the discussion about tongue endurance, the authors argue that the lack of difference between ISSD and TD groups may be due to the difficulty explaining the procedure to the children during data collection but that their results were still consistent with other studies. However, the study they use as an example has found differences in strength with no mention here about endurance. "Nevertheless, the result is consistent with previous study which found difference between typically development and peers with speech impairment with the latter demonstrating decrease tongue strength" | Thanks for this. We have revised the discussion section and referenced appropriately. | See page 16 – 17 |
| In the introduction, on page 10, this sentence seems to be missing an author name at the start: " found a cross-cultural difference on comparing pediatric population measures of tongue strength in Belgian children with those in American children, with the comparison revealing statistical difference on age". | The sentence has been revised as:  ‘In another study, Vanderwegen [23] found a difference in tongue strength among Belgian children, with the comparison revealing statistical differences across age.’ | See page 4 |
| In the introduction, on page 11, please correct the following sentence (repetition): "there is limited evidence on measurement of tongue strength and endurance in non– English-speaking populations is limited". | The sentence has been revised as:  ‘there is limited evidence of the measurement of tongue strength and endurance in non–English-speaking populations.’ | See page 5 |
| And again on page 11, repetition of the word "sex" in the following sentence:  "Is there a difference between participants in terms of child type, sex, age, sex, tongue strength, and tongue endurance?" | The sentence has been revised as:  ‘Are there differences between participants across child type (TD vs. ISSDs), sex, and age on tongue strength and tongue endurance?’ | See page 5 |
| In the methods section, please clarify this sentence: "Because of the participants’ age, verbal motivation was given to Do you mean “encourage their participation for the continuation of the trials". | The sentence has been revised as:  Because of the participants’ age, verbal motivation was given to encourage their continued participation in the trials. | See page 8 |
| In the discussion, please rephrase: "This finding agrees with previous study conducted by which reported differences between children on age ", and correct typos "the demonstrate improve tongue" | The sentence has been revised as:  This finding agrees with those of previous studies reporting differences between children in terms of age [23, 31]. | See page 16 |
